# Caveolin-1 Alters the Pattern of Cytoplasmic Ca^2+^ Oscillations and Ca^2+^-dependent Gene Expression by Enhancing Leukotriene Receptor Desensitization*

**DOI:** 10.1074/jbc.M114.553453

**Published:** 2014-04-22

**Authors:** Yi-Chun Yeh, Ming-Jer Tang, Anant B. Parekh

**Affiliations:** From the ‡ Department of Physiology, Anatomy, and Genetics, University of Oxford, Parks Road, Oxford OX1 3PT, United Kingdom and; the § Department of Physiology, National Cheng Kung University Medical College, Tainan and Department of Life Science, Tunghai University, Taichung 40704, Taiwan

**Keywords:** Calcium Signaling, Caveolin, G Protein-coupled Receptor (GPCR), Gene Transcription, Receptor Desensitization

## Abstract

Cytoplasmic Ca^2+^ oscillations constitute a widespread signaling mode and are often generated in response to stimulation of G protein-coupled receptors that activate phospholipase C. In mast cells, repetitive Ca^2+^ oscillations can be evoked by modest activation of cysteinyl leukotriene type I receptors by the physiological trigger, leukotriene C_4_. The Ca^2+^ oscillations arise from regenerative Ca^2+^ release from inositol 1,4,5-trisphosphate-sensitive stores followed by Ca^2+^ entry through store-operated Ca^2+^ channels, and the latter selectively activate the Ca^2+^-dependent transcription factor NFAT. The cysteinyl leukotriene type I receptors desensitize through negative feedback by protein kinase C, which terminates the oscillatory Ca^2+^ response. Here, we show that the scaffolding protein caveolin-1 has a profound effect on receptor-driven Ca^2+^ signals and downstream gene expression. Overexpression of caveolin-1 increased receptor-phospholipase C coupling, resulting in initially larger Ca^2+^ release transients of longer duration but which then ran down quickly. NFAT-activated gene expression, triggered in response to the Ca^2+^ signal, was also reduced by caveolin-1. Mutagenesis studies revealed that these effects required a functional scaffolding domain within caveolin-1. Mechanistically, the increase in Ca^2+^ release in the presence of caveolin-1 activated protein kinase C, which accelerated homologous desensitization of the leukotriene receptor and thereby terminated the oscillatory Ca^2+^ response. Our results reveal that caveolin-1 is a bimodal regulator of receptor-dependent Ca^2+^ signaling, which fine-tunes the spatial and temporal profile of the Ca^2+^ rise and thereby its ability to activate the NFAT pathway.

## Teaser

**Background:** The scaffolding protein caveolin-1 coordinates membrane signaling clusters, but how this affects Ca^2+^ oscillations is unknown.

**Results:** Caveolin-1 accelerates the rundown of Ca^2+^ oscillations to the agonist leukotriene C_4_, which is prevented by modest inhibition of protein kinase C.

**Conclusion:** Caveolin-1 increases receptor desensitization through Ca^2+^-dependent stimulation of protein kinase C.

**Significance:** The findings reveal how caveolin-1 regulates receptor-dependent Ca^2+^ signaling.

## Introduction

Receptor desensitization is a universal and conserved mechanism that attenuates responses evoked by prolonged stimulation. The kinetics of receptor desensitization vary over orders of magnitude. Kainate receptors desensitize within milliseconds (1), whereas the process develops over hundreds of milliseconds for NMDA receptors (2). By contrast, desensitization of G protein-coupled receptors develops over tens of seconds (3).

In many cell types, moderate stimulation of cell surface receptors that activate the phospholipase C pathway evokes a series of cytoplasmic Ca^2+^ oscillations (4). Information can be encoded in the amplitude, frequency, and spatial profile of the oscillatory signal, leading to activation of selective downstream responses including mitochondrial metabolism, secretion, and gene expression (5).

In mast cells, the activation of cysteinyl leukotriene type I (CysLT1)^3^ receptors with the proinflammatory agonist leukotriene C_4_ (LTC_4_) evokes cytoplasmic Ca^2+^ oscillations. The CysLT1 receptor shows homologous desensitization through which protein kinase C, including the Ca^2+^-dependent α isoform (6), phosphorylates three serine residues on the carboxyl terminus to uncouple the receptor from phospholipase C (7). Acute inhibition of protein kinase C, down-regulation of Ca^2+^-dependent protein kinase C isoforms, or siRNA knockdown of protein kinase Cα all convert the oscillatory Ca^2+^ response into a more sustained Ca^2+^ rise, demonstrating that the oscillatory Ca^2+^ signals are a consequence of reversible receptor desensitization (6), likely reflecting pulsatile increases in InsP_3_.

Reversible receptor desensitization enables phasic Ca^2+^ signals to occur, thereby bypassing the deleterious consequences of a sustained Ca^2+^ rise that include excitotoxicity and Ca^2+^-dependent inhibition of signaling molecules. Mechanisms that control the rate and extent of receptor desensitization will therefore have a profound influence on the spatiotemporal pattern of agonist-evoked Ca^2+^ signals and the subsequent activation of downstream targets. Here we report that the scaffolding protein caveolin-1 enhances desensitization of CysLT1 receptors. The amplitude of Ca^2+^ oscillations is initially increased by caveolin-1, because of enhanced coupling between the receptor and phospholipase C. However, the increased Ca^2+^ mobilization stimulates Ca^2+^-dependent protein kinase C, which then terminates the oscillatory response by accelerating receptor desensitization. Our work identifies caveolin-1 as a bimodal regulator of intracellular Ca^2+^ signals.

## EXPERIMENTAL PROCEDURES

### 

#### 

##### Cell Culture and Transfection

The rat mast cell line RBL-1 was purchased from ATCC (via United Kingdom supplier LCG Standards). For regular maintenance, cells were cultured in Dulbecco's modified Eagle's medium (DMEM) with 10% fetal bovine serum and 1% penicillin/streptomycin at 37 °C with 5% CO_2_ as described (8). For experiments, RBL-1 cells were transfected using the Amaxa system and then incubated overnight in medium without penicillin/streptomycin. Experiments were carried out 24–36 h after transfection.

##### Plasmid Constructs

Wild type caveolin-1 tagged with EGFP was kindly provided by Dr. Suetsugu (University of Tokyo, Japan) (9), and the pleckstrin homology domain linked to GFP (GFP-PHD) was kindly provided by Dr. Meyer (Stanford University) via Addgene. Both caveolin-1-myc-RFP and the tyrosine 14 phospho-inactive form (Y14F caveolin-1-myc-RFP mutant) were kind gifts from Dr. Nabi (University of British Columbia, Canada) (10). Transfection efficiency for these constructs was similar and varied between 30 and 45%.

The scaffolding domain mutant caveolin-1 (F92A,T95A) was generated by using the QuikChange Lightning site-directed mutagenesis kit (Agilent Technologies) with primers list as follows: for mouse caveolin-1 (tagged with EGFP), sense primer 5′-AAGGCCAGCTTCACCACCGCCACTGTGGCAAAATATTGGTTTTACCG-3′ and antisense primer 5′-CGGTAAAACCAATATTTTGCCACAGTGGCGGTGGTGAAGCTGGCCTT-3′; for human caveolin-1 (tagged with RFP), sense primer 5′- AAGGCCAGCTTCACCACCGCCACTGTG*GCG*AAATACTGGTTTTACC-3′ and antisense primer 5′- GGTAAAACCAGTATTTCGCCACAGTGGCGGTGGTGAAGCTGGCCTT-3′.

##### Cytoplasmic Ca^2+^ Measurements

Cells were loaded with Fura-2/AM for 40 min at room temperature in the dark and then washed three times with a solution composed of 145 mm NaCl, 2.8 mm KCl, 2 mm CaCl_2_, 2 mm MgCl_2_, 10 mm d-glucose, and 10 mm HEPES, pH 7.4, with NaOH as described (11). Cells were left for 15 min to allow further de-esterification. Ca^2+^-free solution contained 145 mm NaCl, 2.8 mm KCl, 2 mm MgCl_2_, 10 mm d-glucose, 10 mm HEPES, and 0.1 mm EGTA, pH 7.4, with NaOH. Cytoplasmic Ca^2+^ imaging experiments were carried out using a TILL Photonics system with an IMAGO CCD camera. Cells were excited alternately at 356 and 380 nm, and images were acquired every 2 s. Images were analyzed off line using IGOR Pro for Windows. Ca^2+^ signals are represented at a ratio of 356/380 nm. The experiments illustrated in Fig. 9 were carried out using the imaging system in the laboratory of Dr. Glitsch (Department of Physiology, Anatomy and Genetics, University of Oxford) while repair work was being carried out on our imaging system.

##### Immunocytochemistry and Image Analysis

For immunocytochemistry, cells were transfected with caveolin-1-RFP and FLAG-tagged CysLT1 receptor and then fixed 48 h later with 4% paraformaldehyde and permeabilized with 0.5% Triton X-100. After that, cells were incubated with blocking solution (Thermo Scientific) for 1 h and then incubated with specific primary antibody against FLAG tag (Sigma-Aldrich). Secondary antibody against rabbit IgG was conjugated with Alexa-488 purchased from Invitrogen. Images were obtained by using an Olympus confocal microscope. Relative fluorescence intensity was analyzed using ImageJ software. For cells transfected with GFP-PHD, immunofluorescence images were obtained with a Leica microscope, and the fluorescence intensity was analyzed by ImageJ software. For colocalization studies, confocal images were taken with an FV-1000 confocal microscope (Olympus, Melville, NY), and the colocalization coefficient between two different channels was assessed by the Olympus Fluoview FV1000 system. At least five representative images in each group were used for analysis and 10 different areas on the cell membrane and in the cytosol were selected to obtain Pearson's correlation coefficient.

##### Gene Reporter Assay

24–36 h following transfection with an EGFP-based reporter plasmid under an NFAT promoter, cells were stimulated with LTC_4_ (see text for specific times). The percentage of GFP-positive cells was measured as describe previously (8).

##### Statistical Analysis

All results were expressed as mean ± S.E. A two-tailed Student's *t* test was used to compare differences between two groups, and one-way analysis of variance was used to compare differences when groups numbered three or more (GraphPad Prism). Statistical significance was set at *p* < 0.05, with one, two, and three asterisks denoting *p* < 0.05, 0.01, and 0.001, respectively.

## RESULTS

**FIGURE 1.**
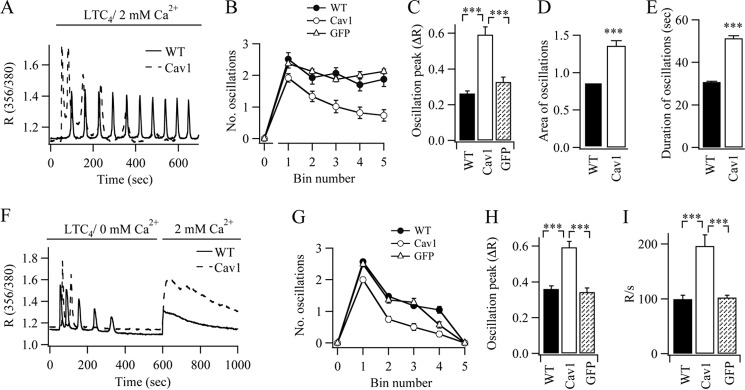
**Caveolin-1 expression increases agonist-evoked Ca^2+^ release from internal stores.** *A*, cytoplasmic Ca^2+^ oscillations to LTC_4_ (applied in the presence of 2 mm external Ca^2+^) are compared between a WT cell and one expressing caveolin-1-GFP (*Cav1*) (*dotted trace*). *B*, the number of oscillations/100-s bin (recording period) is compared for the conditions shown. *GFP* denotes expression of GFP alone. Each data point is the mean of between 21 and 30 cells from three independent experiments. *C–E*, the peak amplitude of the first Ca^2+^ oscillation (*C*), average area of the oscillations (*D*), and mean duration of the oscillations (*E*) are compared among WT (26 cells), GFP-expressing (34 cells), and caveolin-1-GFP-expressing cells (39 cells). For *D* and *E*, the area and duration of each oscillation was measured, and then the data were pooled together. *F*, store-operated Ca^2+^ influx measured following stimulation with LTC_4_ in Ca^2+^-free solution for 600 s followed by readmission of external Ca^2+^ was compared between the two conditions. *G*, the graph compares the rundown of Ca^2+^ oscillations among WT (24 cells), GFP-expressing (29 cells), and caveolin-1-expressing cells (26 cells) when cells were stimulated with LTC_4_ in the absence of external Ca^2+^ as shown in *F. H*, the amplitude of the first Ca^2+^ oscillation, evoked by LTC_4_ in Ca^2+^-free solution, is compared. *I*, the rates of store-operated Ca^2+^ entry, measured by differentiating the Ca^2+^ rise following readmission of 2 mm Ca^2+^ as in *F*, are compared for the conditions shown (each bar denotes >25 cells from three independent experiments).

Endogenous levels of caveolin-1 were virtually undetectable in Western blots from RBL-1 cells (data not shown), so we overexpressed the GFP-tagged protein to study its impact on Ca^2+^ oscillations. In non-transfected (wild type) cells, stimulation with LTC_4_ evoked a series of cytoplasmic Ca^2+^ oscillations (Fig. 1*A*), which decreased slightly over time due to receptor desensitization (Fig. 1*B*) (6). Expression of caveolin-1-GFP substantially altered the pattern of the Ca^2+^ oscillations (Fig. 1*A*, *dotted trace*). The amplitudes of the initial Ca^2+^ oscillations evoked by LTC_4_ were now considerably larger than in non-transfected cells (Fig. 1, *A* and *C*), but the oscillations ran down more quickly and so were fewer in number over a 600 to 700-s recording period (Fig. 1*B*). Analysis of the various oscillatory parameters revealed that the total Ca^2+^ rise associated with each oscillation (area under the spike) was significantly larger in cells expressing caveolin-1-GFP (Fig. 1*D*); this reflected both an increase in the amplitude of each Ca^2+^ oscillation (Fig. 1*C*) as well as an increase in duration (Fig. 1*E*). Cytoplasmic Ca^2+^ during each oscillation was therefore elevated for a longer time in the presence of caveolin-1-GFP.

The effects of caveolin-1-GFP were not mimicked by expression of GFP alone (Fig. 1, *B* and *C*). However, caveolin-1-RFP replicated the marked effects of caveolin-1-GFP on the pattern of Ca^2+^ oscillations (data not shown).

Responses in the presence of external Ca^2+^ reflect both InsP_3_-dependent Ca^2+^ release and Ca^2+^ influx through CRAC channels, the latter being required to replenish the stores with Ca^2+^ in readiness for the next oscillatory cycle. To see which of these processes was affected by caveolin-1, we separated Ca^2+^ release from Ca^2+^ entry by stimulating cells with LTC_4_ in the absence of external Ca^2+^ and then readmitting external Ca^2+^ once the oscillations had run down. Because of the lack of Ca^2+^ influx, Ca^2+^ oscillations decreased in size over time and were lost typically within 400 s after stimulation (the control cell is shown in Fig. 1*F*, and aggregate data are summarized in Fig. 1*G*). Readmission of external Ca^2+^ after 600 s resulted in Ca^2+^ entry through CRAC channels (Fig. 1*F*). Expression of caveolin-1-GFP increased the amplitude of the Ca^2+^ oscillations in Ca^2+^-free solution considerably (Fig. 1*F*; aggregate data are shown in Fig. 1*H*), but these oscillations ran down more quickly than the corresponding control recordings (Fig. 1*G*). Readmission of external Ca^2+^ led to a significantly larger rate of rise of Ca^2+^ (Fig. 1, *F* and *I*), indicating increased store-operated Ca^2+^ influx. Unlike the case of caveolin-1-GFP, expression of GFP alone had no effect on the number of Ca^2+^ oscillations (Fig. 1*G*), the size of the oscillations (Fig. 1*H*), or store-operated Ca^2+^ entry (Fig. 1*I* and Ref. 12).

**FIGURE 2.**
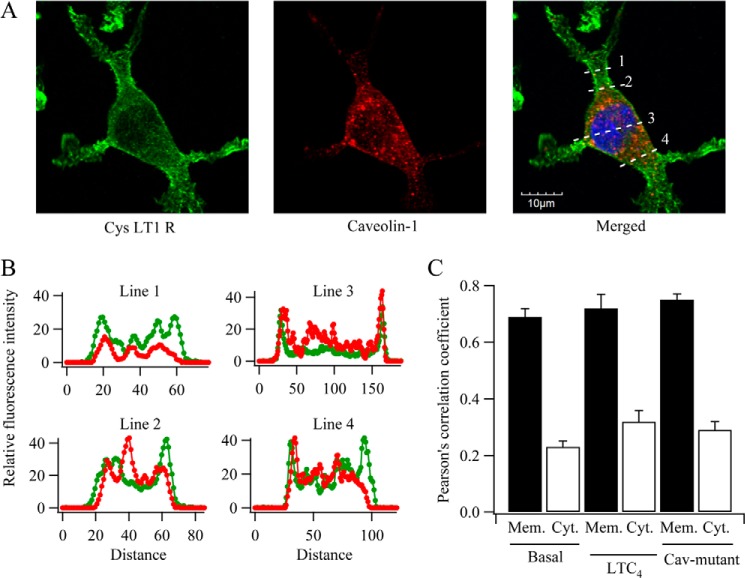
**Subcellular distribution of caveolin-1 and CysLT1 receptors in RBL-1 cells.** Cells were co-transfected with caveolin-1-RFP and FLAG-tagged CysLT1 receptor and then fixed 48 h later. *A*, confocal images for the conditions shown. Line scans are shown in the *merged panel. B*, fluorescence profiles from the line scans are shown. Caveolin-1-RFP distribution is shown in *red*, and FLAG-tagged CysLT1 receptors are in *green. C*, histogram compares Pearson's correlation coefficient for the conditions shown. *CaV-mutant* denotes caveolin-1 with point mutations in the scaffolding domain (see Fig. 4). *Mem*., membrane; *Cyt*., cytosol.

Caveolin-1 increases the interaction between the heterotrimeric GTP-binding protein G_q_ and phospholipase C (13), a mechanism that could explain the increase in amplitude of the Ca^2+^ oscillations. If so, caveolin-1 should be expressed in the plasma membrane. Immunocytochemical studies revealed the presence of both FLAG-tagged CysLT1 receptors and caveolin-1-RFP in the plasma membrane (Fig. 2*A*). A significant fraction of caveolin-1-RFP was also found in the cytoplasm, likely reflecting its contribution to vesicle sorting (14). To test for colocalization, at the level of resolution provided by confocal microscopy, we merged images and measured the subcellular distribution of each protein using line scanning (Fig. 2*A*, *merged panel*). CysLT1 receptor distribution showed two clear peaks, corresponding to plasma membrane at the two edges of the cell (Fig. 2*B*, *green traces*). Although caveolin-1-RFP was present within the cytoplasm, two peaks at the cell periphery were also resolvable, indicating a plasma membrane location. We quantified the extent of overlap of the two proteins using Pearson's correlation coefficient (Fig. 2*C*). Under both basal and stimulated conditions (LTC_4_ exposure for 10 min), there was a much better correlation between FLAG-tagged CysLT1 receptor and caveolin-1-RFP in the membrane than in the cytoplasm, and stimulation did not change the correlation coefficient (Fig. 2*C*).

**FIGURE 3.**
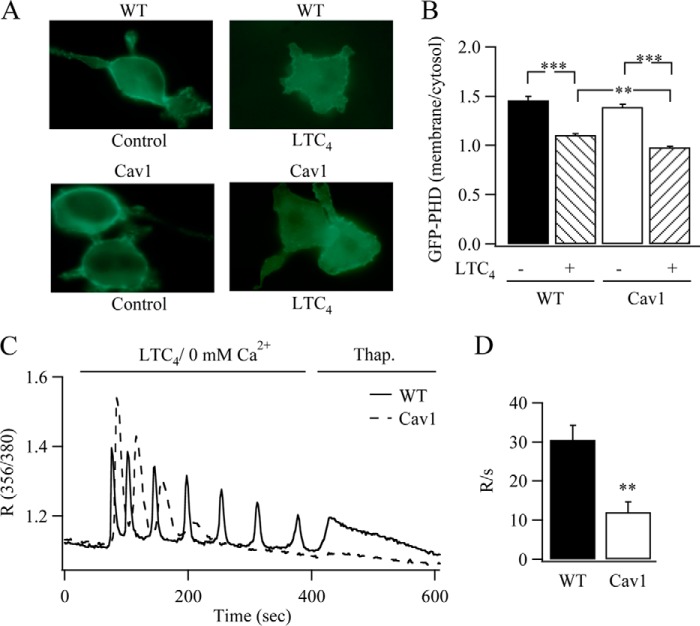
**Receptor-phospholipase C coupling increases in the presence of caveolin-1.** *A*, stimulation with LTC_4_ increases the release of GFP-PHD from the plasma membrane when caveolin-1-RFP (*Cav1*) is present. *B*, aggregate data are summarized (9 and 13 cells for each condition on three separate preparations). *C*, following stimulation with LTC_4_ in Ca^2+^-free solution, the amount of Ca^2+^ remaining in the stores was estimated by application of thapsigargin (*Thap*.; 2 μm). *D*, aggregate data are summarized. The rate of rise of cytoplasmic Ca^2+^ following application of thapsigargin was measured as an indicator of the Ca^2+^ content of the stores. Data represent 44 caveolin-1-GFP-expressing cells and 39 wild type cells from two independent cell preparations. **, *p* < 0.01; ***, *p* < 0.001.

If caveolin-1 increases receptor-phospholipase C coupling, two predictions are that, first, InsP_3_ levels should increase more following stimulation in the presence of caveolin-1 than in wild type cells, and second, less Ca^2+^ should remain within the InsP_3_-sensitive store after the Ca^2+^ oscillations have run down in cells expressing caveolin-1. Using the GFP-PHD construct as a means for monitoring InsP_3_ levels in individual cells (15–17), we found that stimulation with LTC_4_ for 5 min resulted in a modest decrease in the membrane/cytosol ratio of GFP-PHD (decrease of 24.5 ± 1.7%; Fig. 3, *A* and *B*), and this was slightly more pronounced when caveolin-1-RFP was expressed (31.2 ± 1.4%, *p* < 0.05; Fig. 3, *A* and *B*). To test the second prediction, we stimulated cells with LTC_4_ in the absence of external Ca^2+^, and then once the oscillations had stopped, we applied thapsigargin in Ca^2+^-free solution to estimate how much Ca^2+^ remained within the store (Fig. 3*C*). The thapsigargin-mobilizable Ca^2+^ pool was significantly reduced in cells expressing caveolin-1-GFP (Fig. 3*D*, *p* < 0.01).

**FIGURE 4.**
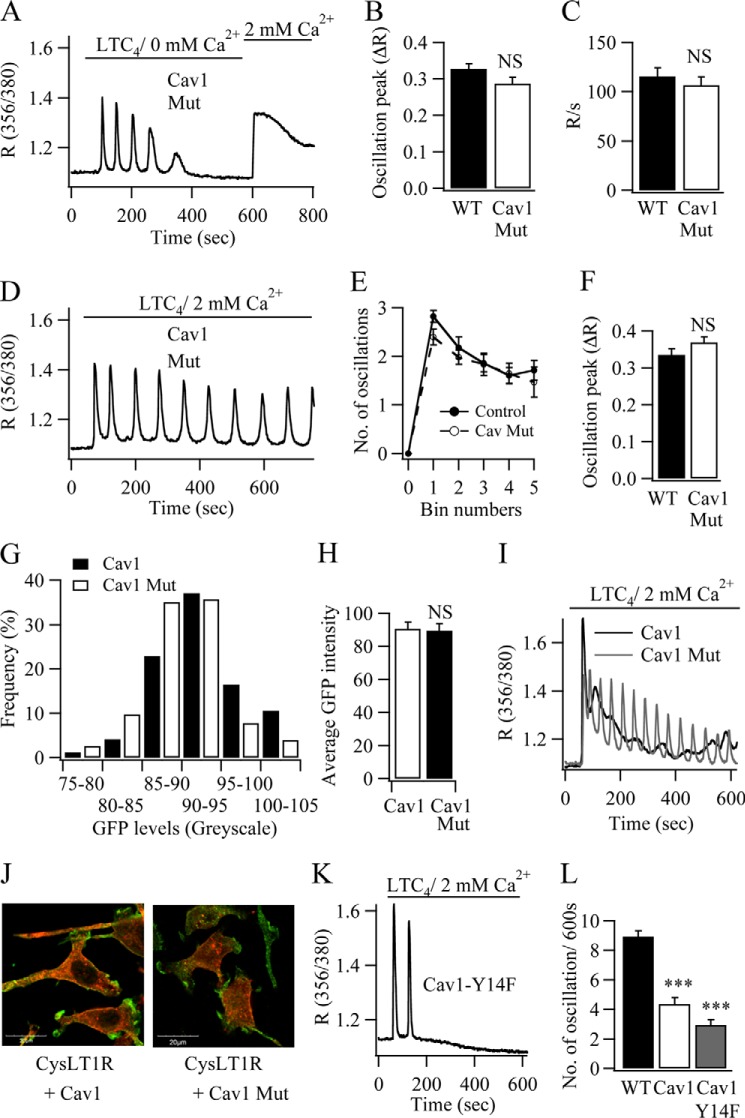
**Mutations within the scaffolding domain abolish the stimulatory effect of caveolin-1-GFP (*Cav1*) on Ca^2+^ release.** *A*, numerous Ca^2+^ oscillations are obtained in Ca^2+^-free solution in response to LTC_4_ when the mutated caveolin-1-GFP protein is expressed. *B* and *C*, the amplitude of the first Ca^2+^ oscillation (*B*) and rate of store-operated Ca^2+^ entry (*C*) are compared between wild type cells and those expressing mutant Cav1-GFP (each *bar* represents between 21 and 31 cells from four independent experiments). *D*, Ca^2+^ oscillations to LTC_4_ in the presence of external Ca^2+^ do not run down in the presence of mutant caveolin-1. *E*, the graph compares the number of oscillations/100-s bin between wild type cells and those expressing the mutant caveolin-1-GFP protein. Each point is between 30 and 60 cells. *F*, the size of the first oscillation in 2 mm external Ca^2+^ is compared for the conditions shown. *G*, the histogram compares the GFP fluorescence for all cells transfected with either caveolin-1-GFP (137 cells) or mutant caveolin-1-GFP (140 cells). *H*, the averaged GFP intensity is compared for the two conditions. *I*, Ca^2+^ responses in two cells that expressed very similar levels of GFP are compared. *J*, *merged confocal images* showing the presence of FLAG-tagged CysLT1 receptors and either Cav1-RFP or mutant Cav1-RFP. *K*, Ca^2+^ oscillations run down quickly when a Cav1 protein is expressed with a mutation in the tyrosine phosphorylation site (Y14F). *L*, aggregate data comparing the number of oscillations over the entire 600-s recording for the conditions described are shown (each *bar* represents the mean of 10 and 17 cells from two independent experiments). *NS*, nonsignificant; ***, *p* < 0.001.

The scaffolding domain of caveolin-1, which involves amino acids between residues 82 and 101, is required for interaction with receptors, G proteins, and other signaling molecules (18, 19). A central core of four amino acids within this region, encompassing ^92^FTVT^95^, is critical for association with G proteins (20). To determine whether this central core was required for regulation of Ca^2+^ signals generated by CysLT1 receptors, we made mutations within the site to see the effect on Ca^2+^ oscillations. Following transfection of a GFP-tagged caveolin-1 construct in which phenylalanine (Phe-92) and threonine (Thr-95) had been mutated to alanines, several Ca^2+^ oscillations were seen in Ca^2+^-free solution (Fig. 4*A*); these were similar in size to those obtained in wild type cells (Fig. 4*B*). The number of oscillations in Ca^2+^-free solution (data not shown) and the rate of rise of the Ca^2+^ signal due to store-operated entry were also not significantly different from control cells (Fig. 4*C*). Cytoplasmic Ca^2+^ oscillations in response to LTC_4_ showed only modest rundown when transfected with the mutated caveolin-1 (Fig. 4*D*), which was not different from wild type cells (Fig. 4*E*). The size of these oscillations was also similar to that in wild type cells (Fig. 4*F*).

We considered the possibility that expression of F92A,T95A caveolin-1-GFP was considerably lower than caveolin-1-GFP, thereby explaining the lack of effect of mutant caveolin-1 on Ca^2+^ oscillations. We therefore compared GFP fluorescence in cells transfected with either caveolin-1-GFP or F92A,T95A caveolin-1-GFP. There was no difference in either the profile of GFP expression between the two groups (Fig. 4*G*) or the averaged GFP fluorescence between the groups (Fig. 4*H*). In Fig. 4*I*, Ca^2+^ signals evoked by LTC_4_ are compared between a cell expressing caveolin-1-GFP and one expressing F92A,T95A caveolin-1-GFP. The cells had almost identical levels of GFP expression (92 and 93 gray scale units, respectively). However, only the presence of caveolin-1-GFP altered the pattern of the Ca^2+^ oscillations. Confocal images showed that both caveolin-1-RFP and F92A,T95A caveolin-1-RFP were expressed at the plasma membrane with FLAG-tagged CysLT1 receptors (Fig. 4*J*). Pearson's correlation coefficient between mutant caveolin-1-RFP and CysLT1 receptors was similar to that seen for caveolin-1-RFP and the receptors (Fig. 2*C*). Collectively, these results show that the scaffolding domain of caveolin-1 is important for the modulation of agonist-evoked Ca^2+^ oscillations.

Phosphorylation of caveolin-1 on tyrosine 14 by Src family kinases potentiates growth factor signaling and is required for internalization of caveolae (21). Expression of an RFP-tagged caveolin-1 construct with a point mutation converting tyrosine to phenylalanine (Y14F) was expressed in the plasma membrane (Fig. 4*J*) and mimicked the effects of caveolin-1-GFP expression on agonist-induced Ca^2+^ oscillations. The initial Ca^2+^ transients were larger (Fig. 4*K*), and fewer oscillations were obtained (Fig. 4*L*). Internalization of caveolin-1 through phosphorylation of tyrosine 14 therefore does not contribute to the effects of caveolin-1 on LTC_4_-driven Ca^2+^ signals.

**FIGURE 5.**
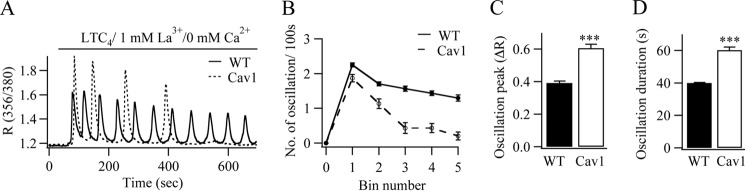
**Ca^2+^ oscillations run down in the presence of caveolin-1-GFP (*Cav1*) under conditions of regenerative Ca^2+^ release.** Cells were stimulated in Ca^2+^-free external solution supplemented with 1 mm La^3+^. *A*, whereas Ca^2+^ oscillations to LTC_4_ in wild type cells are sustained, they run down quickly when caveolin-1-GFP is expressed (*dotted line*). *B*, aggregate data summarizing the number of oscillations per 100 s bin is shown. Each point is the mean between 23 and 41 cells from four independent experiments. *C* and *D*, the amplitude of the first Ca^2+^ oscillation (*C*) and the duration of each oscillation (*D*) are compared. ***, *p* < 0.001.

We designed experiments to identify the mechanism responsible for the accelerated rundown of Ca^2+^ oscillations seen in the presence of caveolin-1. To see whether this was dependent on Ca^2+^ release or Ca^2+^ entry, we stimulated cells in the absence of external Ca^2+^ but with the plasma membrane Ca^2+^ pump blocked with La^3+^. Under these conditions, Ca^2+^ release can no longer be exported out of the cell and instead is sequestrated back into the stores. Ca^2+^ oscillations therefore continue for several minutes, reflecting regenerative Ca^2+^ release in the absence of Ca^2+^ influx (11, 22). Stimulation with LTC_4_ in wild type cells evoked a series of repetitive Ca^2+^ oscillations that decreased slightly in number over time (Fig. 5, *A* and *B*). By contrast, in cells expressing caveolin-1-GFP, larger Ca^2+^ spikes were obtained initially, which then ran down quickly (Fig. 5, *A* and *B*). As with the responses in the presence of external Ca^2+^, the amplitude of the first oscillation (Fig. 5*C*), as well as the duration of the oscillations (Fig. 5*D*), was significantly increased in the presence of caveolin-1-GFP. Rundown of Ca^2+^ oscillations in the presence of caveolin-1 therefore arises from Ca^2+^ release.

**FIGURE 6.**
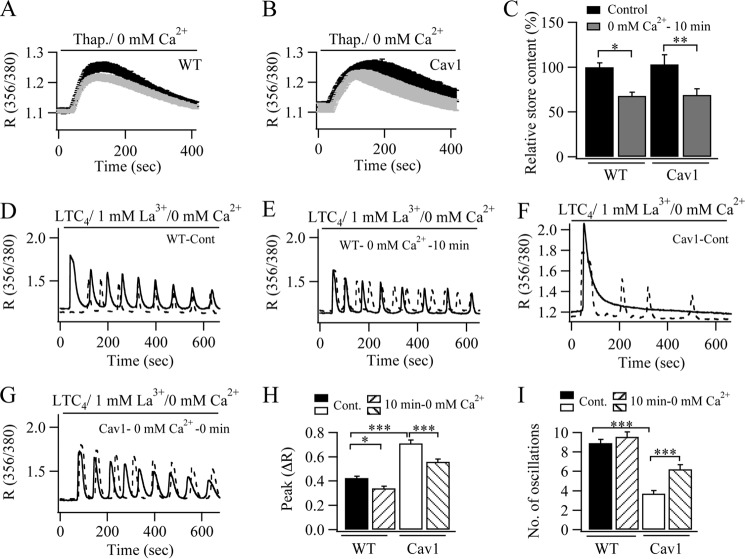
**Reducing the Ca^2+^ content of the stores prior to stimulation reduces the rundown of Ca^2+^ oscillations in caveolin-1-GFP (*Cav1*)- expressing cells.** *A*, preincubation in Ca^2+^-free solution reduces the store Ca^2+^ content, as assessed by the extent of Ca^2+^ release to thapsigargin. The *black trace* is control (∼30 s in Ca^2+^-free solution). The *dotted trace* is the response after 10 min in Ca^2+^-free solution. Each *trace* represents the average of 15–35 cells from four independent experiments. *B*, same as in *A*, but for cells expressing caveolin-1. *C*, aggregate data from several recordings as in *A* and *B* are summarized. *D*, typical oscillatory responses to LTC_4_ obtained in Ca^2+^-free solution containing La^3+^. In each of the *panels D–G*, two cells (*solid line* and *dotted line*) treated the same way but from two different preparations are shown. *E*, typical oscillatory responses after preincubation in Ca^2+^-free solution for 10 min. *F*, oscillatory responses to LTC_4_ in cells expressing caveolin-1. *G*, oscillatory responses to LTC_4_ in caveolin-1-expressing cells after pre-exposure to Ca^2+^-free solution for 10 min. *H*, the amplitude of the first oscillation for each condition is shown. *I*, the average numbers of oscillations obtained over 600 s for each condition are compared. Each *bar* represents between 15–38 cells from three independent experiments. **, *p* < 0.01; ***, *p* < 0.001.

Further evidence that Ca^2+^ release from the stores in caveolin-1-expressing cells contributes to the rundown of the oscillations is shown in Fig. 6. In these experiments, we sought to partially lower the Ca^2+^ content of the stores in order to reduce the size of each Ca^2+^ oscillation upon stimulation. We therefore incubated control (non-transfected) cells in Ca^2+^-free solution for 10 min and found that this was sufficient to reduce the extent of Ca^2+^ release by thapsigargin by ∼ 30% when compared with control cells pre-exposed to Ca^2+^-free solution for just a few seconds prior to stimulation with thapsigargin (Fig. 6, *A* and *C*). We then stimulated cells in Ca^2+^-free solution containing 1 mm La^3+^ to eliminate the increased Ca^2+^ influx due to the reduced store Ca^2+^ content from affecting the oscillatory pattern. Oscillatory Ca^2+^ responses to LTC_4_ were sustained both in cells pretreated with Ca^2+^-free solution acutely (Fig. 6, *D* and *I*) and in those following 10 min of pretreatment (Fig. 6, *E* and *I*), although the size of the oscillations was smaller in the latter case (Fig. 6*H*), reflecting the reduced store Ca^2+^ content. In cells expressing caveolin-1-GFP and incubated in Ca^2+^-free solution for 10 min, the extent of Ca^2+^ release induced by thapsigargin was similar to control cells treated in the same way (Fig. 6, *B* and *C*). Whereas only a few Ca^2+^ oscillations were seen in response to LTC_4_ challenge in caveolin-1-GFP-expressing cells exposed to Ca^2+^-free solution for a few seconds prior to stimulation (Fig. 6, *F* and *I*), preincubation for 10 min with Ca^2+^-free external solution resulted in more prolonged oscillatory Ca^2+^ signals following agonist stimulation (Fig. 6, *G* and *I*). The amplitude of the first Ca^2+^ oscillation was reduced following the 10-min preincubation in Ca^2+^-free solution prior to stimulation (Fig. 6*H*). Hence, lowering the Ca^2+^ content of the stores results in prolonged oscillatory Ca^2+^ signals in the presence of caveolin-1-GFP. These results are consistent with the view that the enhanced Ca^2+^ release normally seen in caveolin-1-expressing cells is responsible for the accelerated rundown of the oscillations.

**FIGURE 7.**
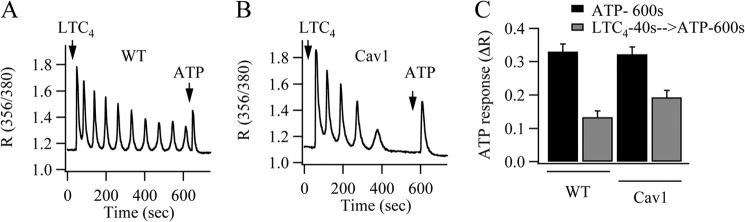
**Rundown of Ca^2+^ oscillations is not associated with inactivation of InsP_3_ receptors.** *A* and *B*, ATP (100 μm) was applied 600 s after stimulation with LTC_4_ (in Ca^2+^-free solution containing 1 mm La^3+^) in either wild type cells (*A*) or in cells expressing caveolin-1-GFP (*Cav1*) (*B*). *C*, aggregate data from several experiments are compared. Each *bar* represents 21–35 cells from two independent experiments. *Black bars* denote responses to ATP in the absence of prior stimulation with LTC_4_.

One way whereby enhanced Ca^2+^ release can increase the rundown of Ca^2+^ oscillations is through Ca^2+^-dependent inactivation of InsP_3_ receptors. However, the Ca^2+^ release transient following phospholipase C-coupled P2Y receptor activation after CysLT1 receptors had been desensitized was slightly larger in caveolin-1-expressing cells (Fig. 7*B*) than in the corresponding controls (Fig. 7*A*; aggregate data are shown in Fig. 7*C*). Inactivation of the InsP_3_ receptor therefore plays little role in the rundown of Ca^2+^ oscillations in the presence of caveolin-1.

**FIGURE 8.**
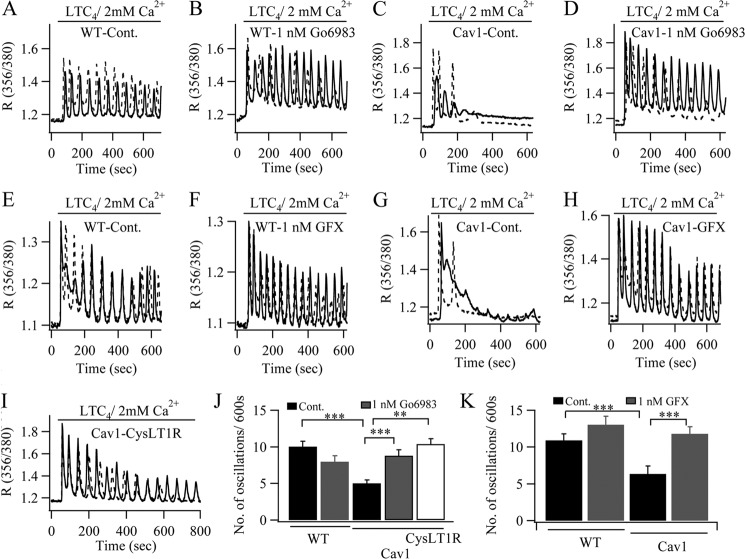
**Modest inhibition of protein kinase C rescues oscillatory Ca^2+^ signaling in cells expressing caveolin-1-GFP (*Cav1*).** In *A–I*, two examples for each condition are shown (*solid* and *dotted lines*). *A*, typical oscillatory responses to LTC_4_ are depicted. *B*, responses from two wild type cells are shown, after pretreatment with Go6983 for 10 min. *C*, responses from two cells overexpressing caveolin-1-GFP are shown. *D*, responses from two caveolin-1-GFP-overexpressing cells pretreated with Go6983 are depicted. *E–H*, same as in *A–D*, but cells were exposed to 1 nm GF109203X instead. *I*, two recordings from cells co-transfected with plasmids encoding caveolin-1-GFP and CysLT1 receptor are shown. *J*, aggregate data from several experiments with Go6983 are summarized. Each *bar* represents between 18 and 25 cells from three independent experiments. *K*, results with GF109203X are compared. Each *bar* denotes 18–26 cells from two independent experiments. **, *p* < 0.01; ***, *p* < 0.001.

**FIGURE 9.**
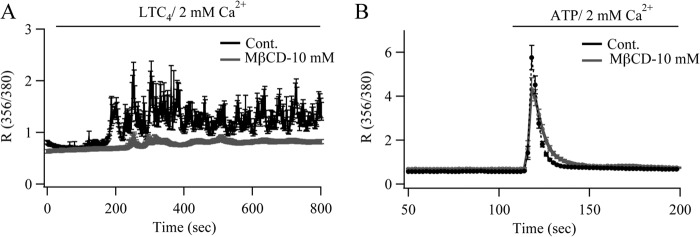
**Disruption of lipid rafts with MβCD abolishes CysLT1 receptor signaling but not P2Y-evoked responses.** *A*, Ca^2+^ signals to LTC_4_ were suppressed following 30 min of pretreatment with MβCD. *B*, MβCD had no clear effect on ATP-driven Ca^2+^ signals. Each *graph* is the average of between 45 and 60 cells from three independent experiments.

We considered that rundown of the Ca^2+^ oscillations was a consequence of the accelerated desensitization of the CysLT1 receptor. These receptors are desensitized following Ca^2+^-dependent protein kinase C-mediated phosphorylation of a series of serine residues on the carboxyl terminus of the receptor, and we had previously found a major role for protein kinase Cα in the desensitization process (6). Increased Ca^2+^ release following caveolin-1-GFP expression would lead to stronger activation of Ca^2+^-dependent protein kinase C isoforms and thus should result in more pronounced receptor desensitization. To test this possibility, we used a low concentration of the protein kinase C inhibitor Go6983 (1 nm) to reduce but not abolish kinase activity, as substantial block of the kinase results in non-oscillatory Ca^2+^ signals (6). The typical oscillatory Ca^2+^ response in wild type cells induced by LTC_4_ stimulation (Fig. 8*A*) was only weakly affected by the low concentration of Go6983 (Fig. 8, *B* and *J*). However, the rapid rundown of Ca^2+^ oscillations in cells expressing caveolin-1-GFP (Fig. 8*C*) was largely prevented by the protein kinase C inhibitor (Fig. 8, *D* and *J*). Identical results were obtained with a structurally different protein kinase C blocker, GF109203X (1 nm; Fig. 8, *E–H* and *K*). Many agonists of G protein-coupled receptors elicit responses by occupying only a fraction of the total receptors. We therefore reasoned that increasing the number of available CysLT1 receptors in the plasma membrane in cells expressing caveolin-1-GFP should lead to an increased likelihood for LTC_4_ to encounter a non-desensitized receptor, which should reduce the rate of rundown of Ca^2+^ oscillations. We therefore transfected cells with plasmids for caveolin-1-GFP and the CysLT1 receptor. Increased expression of CysLT1 receptors significantly prolonged the oscillatory Ca^2+^ response compared with cells transfected with caveolin-1-GFP alone (Fig. 8, *I* and *J*). Despite coupling to phospholipase C via G_q_ proteins, P2Y receptor-driven Ca^2+^ release was unaffected by caveolin-1-GFP expression (ATP responses measured at 600 s in wild type cells and in those expressing caveolin-1-GFP were similar (Fig. 7*C*, *black bars*)). This suggests that P2Y and CysLT1 receptors might couple to phospholipase C differently, with the leukotriene receptor more prominent in caveolin-1-rich domains. Lipid rafts can be disrupted by methyl-β-cyclodextrin (MβCD), a compound that removes cholesterol from the plasma membrane. Treatment with MβCD abolished LTC_4_-dependent Ca^2+^ responses (Fig. 9*A*) but had no significant effect on P2Y-evoked Ca^2+^ signals (Fig. 9*B*). Different agonists thus differ in their sensitivity to regulation by caveolin-1 and lipid rafts.

**FIGURE 10.**
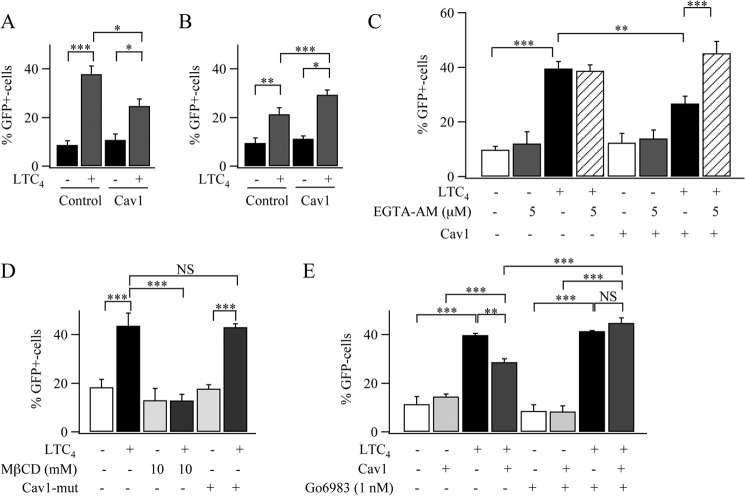
**Caveolin-1 (*Cav1*) regulates Ca^2+^-dependent gene expression.** *A*, expression of a GFP reporter gene under an NFAT promoter is reduced following stimulation with LTC_4_ in caveolin-1-RFP-expressing cells compared with control cells. *Control* denotes cells transfected only with GFP under the NFAT promoter. 24 h after NFAT-GFP transfection, cells were stimulated overnight with 160 nm LTC_4_. *B*, Cav1 does not impair LTC_4_-induced gene expression when slow inactivation is reduced. Here, cells were stimulated with LTC_4_ in Ca^2+^-free solution for 8 min, and then external Ca^2+^ was readmitted for 5 min before cells were placed in culture medium and left in the incubator overnight. *C*, aggregate data for the various conditions are compared. Stimulation with LTC_4_ was carried out as in *A. D*, aggregate data for the conditions shown are compared. Stimulation with LTC_4_ was as in *A. E*, the effects of a low concentration of Go6983 on gene expression induced by LTC_4_ is compared between control cells and those expressing Cav1-RFP. All data are aggregates from three independent experiments with between 50 and 80 cells from each experiment. Stimulation with LTC_4_ was as in *A. NS*, not significant; **, *p* < 0.01; ***, *p* < 0.001.

To see whether the altered pattern of Ca^2+^ signaling by caveolin-1 had functional relevance, we measured Ca^2+^-dependent gene expression using a GFP construct under a promoter driven by the Ca^2+^-dependent transcription factor NFAT (8, 23). In non-stimulated cells, expression of GFP was low (Fig. 10*A*), but it increased ∼4-fold after LTC_4_ was added to the culture medium. Basal gene expression was also low in caveolin-1-RFP-expressing cells, but stimulation resulted in a relatively weaker rise (∼ 2.5 fold, Fig. 10*A*; *p* < 0.01). Because NFAT activation is tightly linked to local Ca^2+^ entry through CRAC channels following physiological levels of stimulation in RBL cells (8, 24), we hypothesized that the larger size and longer duration of the Ca^2+^ release transients in the presence of caveolin-1 (Fig. 1, *C* and *E*) increased Ca^2+^-dependent slow inactivation of CRAC channels (25, 26) and thereby reduced NFAT-dependent gene expression. One way to reduce Ca^2+^-dependent slow inactivation of CRAC channels is to use a different stimulation protocol. Stimulation with LTC_4_ for 10 min in the absence of external Ca^2+^ fails to activate gene expression despite evoking several Ca^2+^ oscillations (8). Readmission of external Ca^2+^, a few minutes after the oscillations have run down, allows for recovery from slow inactivation. Using this protocol, we found that expression of caveolin-1-RFP now failed to reduce NFAT-dependent gene expression (Fig. 10*B*). In fact, expression increased somewhat, in accordance with the increase in store-operated Ca^2+^ entry that arises from the more extensive store depletion (Fig. 1*F*). Because Ca^2+^-dependent slow inactivation requires a rise in bulk Ca^2+^, it can be prevented by the slow Ca^2+^ chelator EGTA (25, 26). We therefore reduced the Ca^2+^ rise by loading the cytoplasm with EGTA. EGTA had no inhibitory effect on LTC_4_-induced gene expression in control cells (Fig. 10*C*), but it prevented the reduction in gene expression seen in the presence of caveolin-1-RFP (Fig. 10*C*). The reduction in LTC_4_-driven gene expression in caveolin-1-RFP-expressing cells was not seen when F92A,T95A caveolin-1-RFP was expressed instead (Fig. 10*D*). Gene expression was also impaired after lipid raft disruption with MβCD (Fig. 10*D*). The reduction in gene expression to LTC_4_ in cells expressing caveolin-1-RFP was prevented by pretreating cells with 1 nm Go6983 (Fig. 10*E*), a concentration that rescued repetitive Ca^2+^ signaling to agonist (Fig. 8).

## DISCUSSION

Caveolin-1 is a conserved plasma membrane scaffolding protein that facilitates interaction between signaling molecules within subcompartments of the membrane. One such interaction involves enhanced coupling between G_q_ and phospholipase C, thereby generating larger increases in InsP_3_ (13). Our data add a new aspect to this role for caveolin-1, namely in triggering receptor desensitization and thus terminating Ca^2+^-dependent responses following physiological levels of stimulation.

Stimulation of CysLT1 receptors with LTC_4_ leads to repetitive Ca^2+^ oscillations, which reflect regenerative Ca^2+^ release followed by transient Ca^2+^ entry through CRAC channels (11). The Ca^2+^ oscillations can be converted into a more prolonged non-oscillatory Ca^2+^ rise by interfering with protein kinase C activity (6). Protein kinase C triggers CysLT1 receptor desensitization through phosphorylation of three serine residues on the carboxyl terminus of the receptor (7). Overexpression of caveolin-1 resulted in Ca^2+^ oscillations with larger amplitude and greater duration, as expected from increased G_q_-phospholipase C coupling. However, the oscillations ran down more quickly and Ca^2+^-dependent gene expression was reduced following overexpression of caveolin-1. The rundown was not due to compromised store refilling or inactivation of the InsP_3_ receptors. Rather, the increased Ca^2+^ release in the presence of caveolin-1 led to stronger Ca^2+^-dependent activation of protein kinase C, which resulted in increased leukotriene receptor desensitization. Partial block of protein kinase C reversed the effects of caveolin-1 on oscillation amplitude, duration, rundown, and gene expression. The increase in size and duration of Ca^2+^ release in the presence of caveolin-1 would lead to enhanced Ca^2+^-dependent inactivation of CRAC channels (25, 26). Because Ca^2+^ microdomains near these channels activate gene expression, larger or prolonged Ca^2+^ release impairs transcription by reducing CRAC channel activity.

CysLT1 receptors and caveolin-1 are co-expressed in various tissues, suggesting that the interaction we have described here might occur in other cell types as well. Airway smooth muscle expresses both CysLT1 receptors (27) and caveolin-1 (28), as do macrophages (29, 30), human umbilical vein endothelial cells (31, 32), and human colon, pancreas, and spleen (33, 34).

Our results reveal a novel mechanism for cytsteinyl leukotriene receptor desensitization involving caveolin-1. Enhanced Ca^2+^ release due to increased coupling between the receptor and phospholipase C both activates Ca^2+^-dependent protein kinase C, which leads to pronounced receptor desensitization, and accelerates Ca^2+^-dependent slow inactivation of CRAC channels. Activation of this pathway likely involves subcompartments within the membrane, as P2Y receptor-dependent Ca^2+^ release was unaffected by caveolin-1. By regulating desensitization, caveolin-1 is therefore an important determinant of the duration of receptor stimulation and thus of subsequent Ca^2+^-dependent downstream signaling.

## Footnotes

3 The abbreviations used are:

CysLT1: cysteinyl leukotriene type I
LTC_4_: leukotriene C_4_
InsP_3_: inositol 1,4,5-trisphosphate
PHD: pleckstrin homology domain
RFP: red fluorescent protein
NFAT: nuclear factor of activated T-cells
CRAC: Ca2^+^-activated Ca2^+^
MβCD: methyl-β-cyclodextrin.
